# Metallothionein 2A (MT2A) controls cell proliferation and liver metastasis by controlling the MST1/LATS2/YAP1 signaling pathway in colorectal cancer

**DOI:** 10.1186/s12935-022-02623-w

**Published:** 2022-05-31

**Authors:** Xi Liu, Jun Quan, Zhaolong Shen, Zequn Zhang, Zhijian Chen, Liang Li, Xiaorong Li, Gui Hu, Xiaofeng Deng

**Affiliations:** 1 Department of Gastrointestinal Surgery, The Third Xiangya Hospital of Central South University, Tongzipo Road, Changsha, 410013 Hunan People’s Republic of China; 2 Department of General Surgery, The Second Xiangya Hospital of Central South University, Renmin Road, Changsha, 410000 Hunan People’s Republic of China

**Keywords:** Colorectal cancer, Liver metastasis, Metallothionein, MT2A, Hippo signaling pathway

## Abstract

**Background:**

Colorectal cancer (CRC) is one of the three major cancers in the world and is the cancer with the most liver metastasis. The present study aimed to investigate the role of metallothionein 2A (MT2A) in the modulation of CRC cell proliferation and liver metastasis, as well as its molecular mechanisms.

**Methods:**

The expression profile of metallothionein 2A (MT2A) in colorectal cancer retrieved from TCGA, GEO and Oncomine database. The biological effect of MT2A overexpression was investigated mainly involving cell proliferation and migration in CRC cells as well as growth and metastasis in CRC animal models. To explore the specific mechanism of MT2A metastasis in CRC, transcriptome sequencing was used to compare the overall expression difference between the control group and the MT2A overexpression group.

**Results:**

Metallothionein 2A (MT2A) was downregulated in the tumor tissues of patients with CRC compared to adjacent normal tissues and was related to the tumor M stage of patients. MT2A overexpression inhibited CRC cell proliferation and migration in cells, as well as growth and metastasis in CRC animal models. While knockdown of MT2A had the opposite effect in cells. Western blotting confirmed that MT2A overexpression promoted the phosphorylation of MST1, LAST2 and YAP1, thereby inhibiting the Hippo signaling pathway. Additionally, specific inhibitors of MST1/2 inhibited MT2A overexpression-mediated phosphorylation and relieved the inhibition of the Hippo signaling pathway, thus promoting cell proliferation. Immunohistochemistry in subcutaneous grafts and liver metastases further confirmed this result.

**Conclusions:**

Our results suggested that MT2A is involved in CRC growth and liver metastasis. Therefore, MT2A and MST1 may be potential therapeutic targets for patients with CRC, especially those with liver metastases.

**Supplementary Information:**

The online version contains supplementary material available at 10.1186/s12935-022-02623-w.

## Background

At present, colorectal cancer (CRC) is one of the three major malignant tumors in the world, accounting for 1 in every 10 cancer patients, and its mortality rate is second only to lung cancer. It is estimated that 1,880,725 new cases of colorectal cancer and 915,880 deaths will occur in 2020 [1]. The rich blood supply of the liver provides an abundant environment for tumor metastases, which is the most common metastatic site of CRC. Approximately 50% of CRC patients have liver metastasis (CRLM) at the time of diagnosis of primary tumors. Liver resection is the preferred treatment for CRLM with a 5-year survival rate of 20–50%, which is location-dependent (liver metastasis on the right side of primary CRC has a worse prognosis than that on the left side), and it is consistent with the invasiveness and poor prognosis mediated by mutation of some genes (for example, BRAF-V600E mutant CRLM with aggressive biological behavior is more likely to occur on the right side of primary CRC) [2]. In addition, only 15–20% of patients with CRLM receive liver resection surgery [3]. Although liver transplantation is another option, it is constrained by strict inclusion and exclusion criteria as well as a shortage of donors and unresolved disputes. Therefore, it is of great clinical significance to explore the mechanism of CRLM and identify promising diagnostic and therapeutic targets for CRLM.

Metallothionein (MT) is a cysteine-rich protein that plays an important role in metal homeostasis, heavy metal toxicity, DNA damage and oxidative stress protection. The abnormal expression of MT genes is observed in a variety of cancers and is associated with tumor formation, metastasis, drug resistance and poor prognosis. Differential expression of specific MT isoforms can be used for the diagnosis and treatment of cancers [4]. For example, MT1E expression is downregulated in hepatocellular carcinoma (HCC), and MT1E induces apoptosis of HCC cells and inhibits metastasis [5]. Similarly, high expression of MTF2 in HCC is closely related to clinical characteristics and prognosis. In vitro experimental results have shown that MTF2 significantly promotes the growth, migration and invasion of HCC cells, and MTF2 overexpression promotes growth and epithelial-mesenchymal transformation by promoting Snail transcription [6]. The expression level of metallothionein 2A (MT2A), also known as MT2, is associated with tumor types. For example, MT2A is downregulated in gastric cancer [7] and is related to the chemotherapy sensitivity of gastric cancer cells to docetaxel [8]. Moreover, MT2A exerts its anti-gastric cancer effect by combining with MZF1 to target NFKBIA [9]. However, MT2A is highly expressed in esophageal squamous cell carcinoma (ESCC) cells and promotes ESCC cell migration and invasion by regulating the expression and secretion of IGFBP2, thus influencing the NFκB, Akt and Erk signaling pathways [10]. Similarly, MT2A depletion is associated with decreased expression of TGF-α and MMP-9, and MT2A silencing reduces the migration and invasion activities of mucoepidermoid carcinoma [11].

In addition, the differential expression of MT2A also depends on tumor differentiation status as well as other environmental stimuli and gene mutations [4]. In CRC, studies have shown that MT2A expression is both upregulated and downregulated [12–14], but the specific role and molecular mechanism of MT2A in CRC remain unclear. To explore the underlying differences, we identified that MT2A was significantly downregulated in CRC in TCGA, GSE and Oncomine databases, and we confirmed this result in 65 pairs of cancer and adjacent tissues in patients with CRC. More importantly, we found that MT2A expression was associated with the tumor M and N phases but not the T phase. Further experiments showed that MT2A overexpression inhibited the proliferation and metastasis of HCT8 and HCT116 cells, and it was confirmed that overexpression of MT2A inhibited liver metastasis in animal models with hepatic metastasis established by subsplenic injection of HCT8 cells. To explore the specific mechanism, transcriptome sequencing was used to compare the overall expression difference of MT2A between the MT2A-overexpressing and control groups, and it was found that MT2A overexpression was significantly correlated with the Hippo signaling pathway. Studies have shown that the key molecules of Hippo signaling, namely, MST1, LATS2 and YAP1, activate and drive cancer cell survival, proliferation, invasive migration and metastasis in a variety of tumors [15]. Our in vitro and in vivo experiments also confirmed that MT2A overexpression promoted the expression of phosphorylated MST1, LATS2 and YAP1, thus inhibiting the Hippo signaling pathway, while XMU-MP-1 (a specific inhibitor of MST1/2) relieved the inhibitory effect of MT2A on Hippo signaling. Our results suggested that the downregulation of MT2A in CRC promotes CRC cell proliferation and liver metastasis through the MST1/LATS2/YAP1 signaling pathway, suggesting that MT2A may be a therapeutic target for CRC, especially CRLM.

## Materials and methods

### Cell lines and human samples

The HCT8 and HCT116 CRC cell lines were purchased from ZSBIO (China; possessing cell identifications). Both cell lines were cultured with RPMI-1640 supplemented with 10% FBS and placed in an incubator at 5% CO_2_ and 37 °C. Surgical samples were collected from 65 patients with CRC in the Department of General Surgery of the Second Xiangya Hospital of Central South University. After treatment, the samples were wrapped in paraffin and preserved. This study was approved by the Ethics Committee of The Second Xiangya Hospital of Central South University, and informed consent was obtained from the patients.

### RNA extraction and quantitative real-time PCR (qRT–PCR)

Total RNA was extracted using TRIzol according to the manufacturer's instructions, and the concentration of total RNA was measured using a NanoDrop. RNA (1000 ng) from each sample was used for reverse transcription into cDNA using a RevertAid™ First Strand cDNA Synthesis Kit (Thermo Scientific Co., Ltd.) according to the manufacturer’s instructions. Finally, cDNA was mixed with primers and 2X SYBR Green Fast qPCR Mix (ABclonal, Inc.), and the mRNA levels were detected using a qPCR instrument. The relative expression content of the target gene was normalized to GAPDH. The primers spanned exons for each gene. The following primers were purchased from Sangon Biotech Co., Ltd.: MT2A forward, 5′-ATGGATCCCAACTGCTCCTG-3′; MT2A reverse, 5′-AGCAGCAGCTTTTCTTGCAG-3′; GAPDH forward, 5′-GTCAAGGCTGAGAACGGGAA-3′; and GAPDH reverse, 5′-AAATGAGCCCCAGCCTTCTC-3′.

### Cell proliferation rate and colony formation

To detect the proliferation ability of cells, the experimental cells of each group were prepared into single-cell suspensions. Cells were seeded into 96-well plates and cultured for the indicated days, and the cell proliferation rate was then determined by a CCK-8 kit (Beyotime). For the colony formation assay, the single-cell suspension was plated on a flat 6-well plate at a density of 2000 cells per well. After implantation, colony formation was monitored using an inverted microscope at 3, 7 and 10 days. After the colony formation experiment was completed, cells were washed with PBS and fixed with 4% paraformaldehyde for 1 h. Cells were washed with PBS, and cells were then stained with crystal violet.

### EdU assay

The BeyoClick™ EdU Cell Proliferation Kit with DAB (Beyotime Biotechnology Co., Ltd.) was used to detect the proliferation of individual cells. After cells were cultured in 24-well plates for 24 h, they were washed with PBS, and EdU was then added followed by incubation at 37 °C for 2 h. Cells were then washed three times with PBS and permeabilized with 0.25% Triton X-100 (Genview, China). Nuclei were stained with DAPI, and cells were examined with a fluorescence microscope (Olympus Inc., USA).

### Transwell assay

To detect the migration ability of cells, MT2A-overexpressing cells and control cells were prepared into single-cell suspensions. Single-cell suspensions were plated in the upper Transwell chamber at a density of 5 × 10^4^ or 1 × 10^4^ cells per well as indicated, and 500 μL of medium containing 10% fetal bovine serum was added to the bottom chamber. After 24 h of culture, cells were fixed with 70% methanol for 1 h and stained with crystal violet. The number of migrating cells was detected by inverted microscopy.

### Western blot (WB) assay

Cells overexpressing MT2A and control cells were collected and lysed with RIPA buffer containing protease inhibitors and phosphatase inhibitors for protein extraction. The total protein concentration was determined using a Pierce™ BCA protein assay kit (Thermo Scientific Co., Ltd.). SDS loading buffer (5 ×) was added to the lysis mixture containing 50 μg of total protein and heated at 95 °C for 10 min. Electrophoresis was performed with a 12% or 10% SDS–polyacrylamide gel followed by transfer to a 0.2 μm nitrocellulose membrane. After blocking with 5% bovine serum albumin at room temperature for 1 h, the membrane was incubated with primary antibodies according to the manufacturers’ instructions. The antibodies used for WB analysis are listed in Table 1.

**Table 1:** Antibodies for WB analysis and IHC staining

Antibody	Reagent	Product number	Assay
MT2A Rabbit pAb	Abcam	Ab192385	WB
MT2A Rabbit pAb	Abclonal	A2018	IHC
MST1 Rabbit pAb	Abclonal	A12963	WB
pMST1 Rabbit pAb	CST	49332	WB, IHC
LATS2 Rabbit pAb	Abclonal	A16249	WB
pLATS2 Rabbit pAb	Abclonal	AP0904	WB
YAP1 Rabbit pAb	Abclonal	A19134	WB
pYAP1 Rabbit pAb	CST	13008	WB, IHC
GAPDH Rabbit pAb	Abclonal	Ab9485	WB

### Hematoxylin–Eosin (H&E) staining

Paraffin sections of liver metastases established by MT2A-overexpressing cells (n = 4) and control cells (n = 4) were prepared and stained with hematoxylin and eosin for 10 and 3 min, respectively. After dehydration with ethanol, the sections of tissue samples were dried and sealed with neutral resin for preservation. Finally, an inverted microscope was used for observation and to acquire images.

### Immunohistochemistry (IHC) staining

Paraffin sections of patient samples, subcutaneous grafts and liver metastases established with MT2A-overexpressing cells and control cells were prepared. For patient samples, two pathologists independently scored the MT2A staining, and cases with positive cells ≥ 25% were considered positive whereas other cases were considered negative. When divergence happens, they would discuss it. After treatment with IHC antigen repair solution, endogenous peroxidase and biotin were inactivated by 0.3% hydrogen peroxide for 30 min, and 5% BSA was used for blocking for 30 min. Antibodies were incubated according to the antibody instructions (Table 1) followed by DAB staining for 4 min. Neutral resin was used to seal the sections for preservation. An inverted microscope was used for observation and to obtain images.

### MT2A-lentivirus

For overexpression of MT2A in vitro, lentivirus vectors harboring full-length MT2A were designed, and an empty vector was used as the negative control. The lentivirus vectors were purchased from GeneChem Co., Ltd. (China). The multiplicity of infection was 40, and the period was 7 days. The efficiency was verified by qRT-PCR and Western blots.

### MT2A siRNA transfection

MT2A siRNA was designed and synthesized by RiboBio (China). Transfection (100 pmol/mL) was performed using Lipofectamine™ RNAiMAX reagent. The efficiency was verified by determining MT2A mRNA expression in HCT8 cells.

### Immunofluorescence staining

HCT8 Cells were grown on cover glasses for 24 h. After treated as indicated, cells were fixed, permeabilized, blocking by 3% bovine serum albumin. Then cells were ready to incubate with rabbit anti-YAP1 antibody overnight. The next day, cells were incubated with donkey anti-rabbit Alexa Fluo594 (Invitrogen) for 45 min at 37 °C. Cells were rinsed and mounted on cover glasses with DAPI (Invitrogen). Finally, signal was captured by laser confocal microscopy (Zeiss Inc., Germany).

### Subcutaneous graft CRC and liver metastasis models

For the subcutaneous model, MT2A-overexpressing cells and control cells were prepared as single-cell suspensions. Four-week-old female BALB/C nude were selected for this experiment. After the skin of the shoulder and back of nude mice were disinfected with complexing iodine, a suspension containing 2 × 10^6^ cells was injected under the skin to construct a subcutaneously transplanted tumor model. Tumor size was observed and recorded every 5 days, and mice were sacrificed 28 days later.

For the liver metastasis model, anesthetized mice were subjected to a laparotomy. A cell suspension containing 2 × 10^6^ cells was then injected under the capsule of the lower pole of the spleen, and the incision was sutured. After 4 weeks, the nude mice were sacrificed, and the number of metastatic nodules on the surface and inside of the liver were counted by the naked eye and microscope, respectively, and the metastatic nodules were confirmed by H&E staining.

### Transcriptome sequencing

RNA samples were extracted from MT2A-overexpressing cells and control cells and sent to Novogene, China, for quality inspection and transcriptome sequencing. The sequencing libraries were generated using the NEBNext^®^ Ultra™ RNA Library Prep Kit. R Studio software was used for differential expression analysis and GSEA of the normalized sequencing data.

### Human phospho-kinase array

A Proteome Profiler™ Array Human Phosphokinase Array Kit (RnDSystems, America) was used to detect protein phosphorylation levels according to product specifications. After the membranes were washed with blocking buffer, cell lysate was added and incubated at 4 °C overnight. Membranes were washed three times with PBS, and the membranes were then incubated with the antibody cocktail at room temperature for 2 h. After washing the membranes, streptavidin-HRP was added and incubated for 30 min. After washing again, Chemi Reagent Mix was used to expose the membranes. The membrane was divided into two parts to improve sensitivity and reduce cross-reaction.

### Statistical analysis

All statistical analyses were performed using Prism software (GraphPad Prism 8). A two-tailed Student’s t test was used to assess significant differences between two groups. For three or more groups, one-way ANOVA was used. The expression of MT2A with clinical factors was analyzing using χ^2^ test or Fisher’s exact test, as appropriate. We used mean and SEM for description of in vitro data, and mean and SD for in vivo data. P values less than 0.05 were considered statistically significant.

## Results

### MT2A is downregulated in CRC and correlates with clinicopathological characteristics

**Fig. 1:**
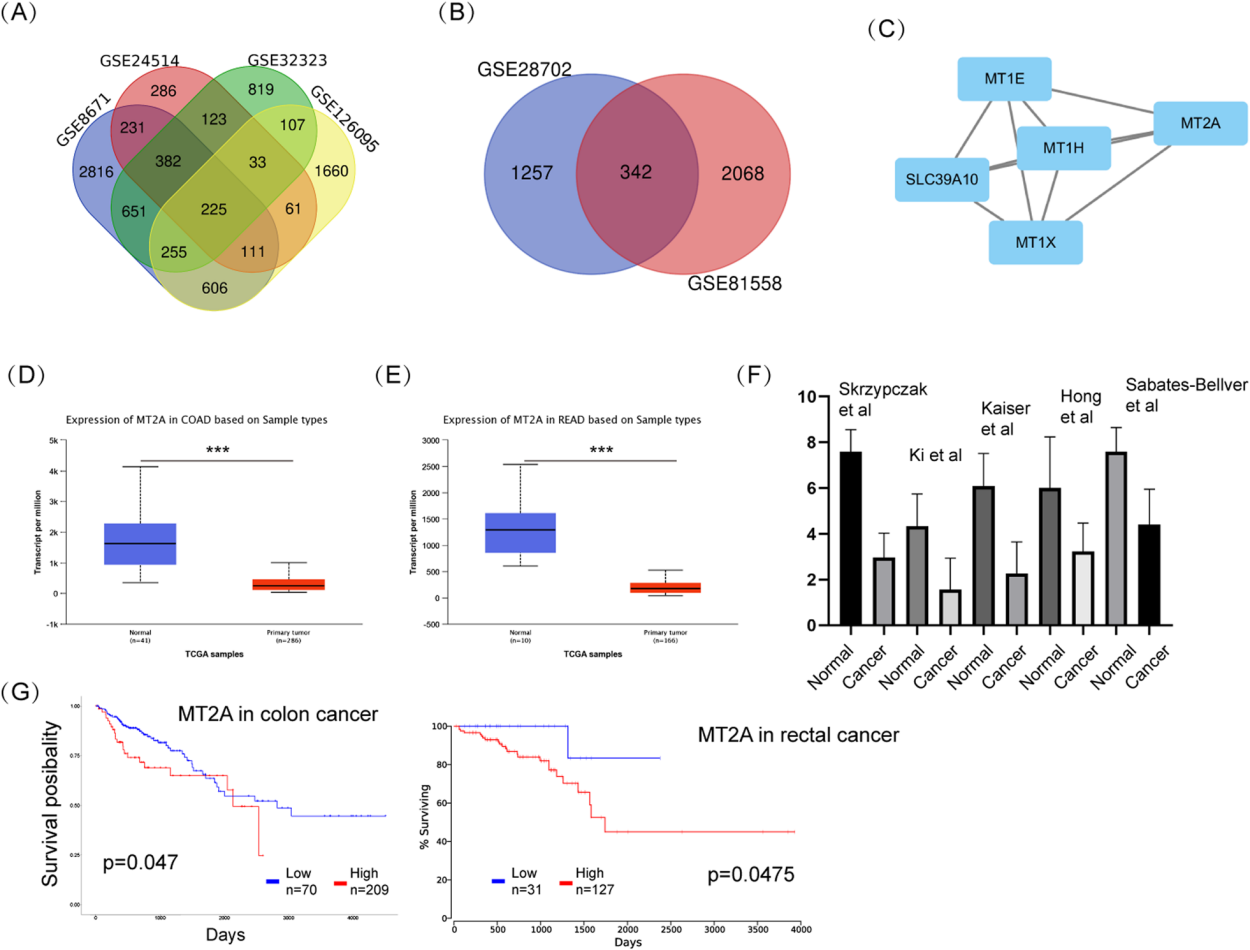
MT2A is downregulated in CRC samples. **A** Four GSE datasets (GSE8671, GSE24514, GSE32323 and GSE126095), which involved data of primary colorectal cancer and paracancerous tissues, were utilized. With a cutoff of p < 0.01 and |log_2_fold change|> 1, 225 genes were found to be commonly altered in the four datasets. **B** Two GSE datasets (GSE28702 and GSE81558), were used to compare the gene expression between primary and liver metastasis tumors, and 342 genes were found to be commonly altered. **C** The MCODE plug-in APP of Cytoscape was used to select the significant module in the PPI network and generate the most likely potential function cluster. Five genes, namely, MT1E, MT2A, MT1H, MT1X and SLC39A10, were included in the predicted cluster. **D** TCGA data from COAD were used to examine the expression of MT2A in colon cancer, and MT2A was significantly downregulated in cancer tissues compared to normal tissues. **E** TCGA data from READ were used to examine the expression of MT2A in rectal cancer, and MT2A was significantly downregulated in cancer tissues compared to normal tissues. **F** Five Oncomine datasets from Skrzypczak et al., Ki et al., Kaiser et al., Hong et al. and Sabates-Bellver et al. were selected, and all showed that MT2A was downregulated in cancer tissues. **G** Clinical data of patients with colon and rectal cancer were obtained from TCGA, and higher expression of MT2A indicated poor prognosis in both colon and rectal cancer patients. ***p < 0.001

To examine the expression of MT2A in CRC samples, we retrieved data from TCGA and GSE datasets as well as the Oncomine database. Four GSE datasets (GSE8671, GSE24514, GSE32323 and GSE126095) were selected to examine the differentially expressed genes between primary tumor and normal colorectal tissues. Using a cutoff of p < 0.01 and |log_2_fold change|> 1, 225 genes were commonly varied in these datasets (Fig. 1A). Two datasets (GSE28702 and GSE81558) were then selected to compare the differentiated genes between primary and liver metastasis tissues, resulting in 342 genes (Fig. 1B). The MCODE plug-in APP of Cytoscape was used to select the significant module in the protein–protein interaction (PPI) network and generate the most likely potential function cluster, which comprised 5 genes, including MT2A (Fig. 1C). To further verify the expression of MT2A in CRC, TCGA data were used and analyzed by UALCAN online tool (http://ualcan.path.uab.edu), and significant downregulation of MT2A was observed in both colon cancer and rectal cancer (Fig. 1D, E). In addition, five Oncomine datasets showed the same trend of MT2A expression in CRC (Fig. 1F). Regarding prognosis, lower expression indicated an improved survival of CRC patients (Fig. 1G). These results showed that MT2A is significantly downregulated in CRC tissues.

**Fig. 2:**
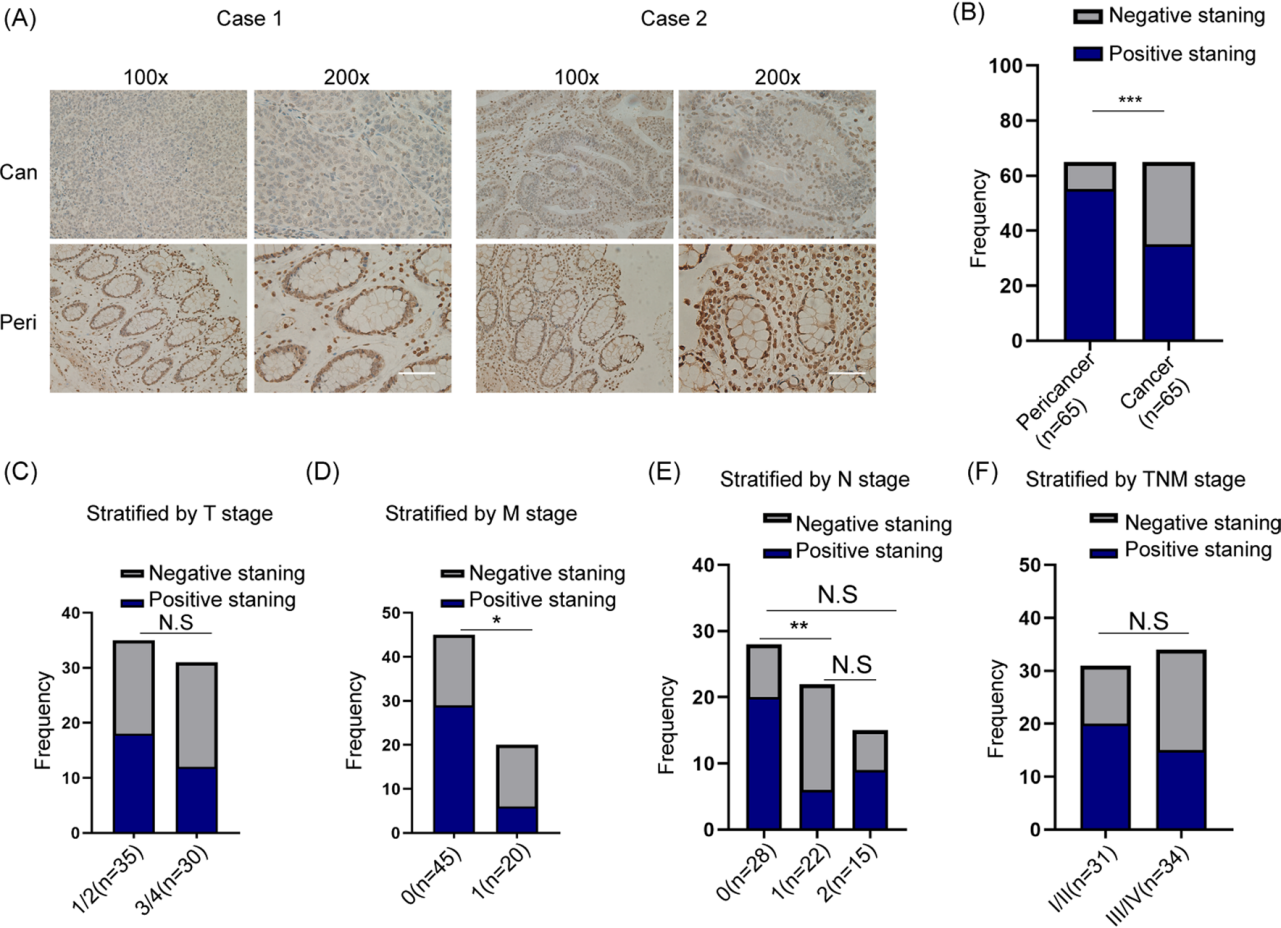
Expression of MT2A is correlated with clinicopathological characteristics in CRC. **A** CRC patients who underwent surgery were included in our analysis, and IHC staining was used to compare the expression of MT2A in cancer and paracancerous tissues. **B** The staining score of MT2A was analyzed by two pathologists and expressed as frequency. The results showed that MT2A in cancer tissues was significantly decreased compared to paracancerous tissues (n = 65). **C** When stratified by T stage, no significant difference was observed between stages 1/2 (n = 35) and stage 3/4 (n = 30). **D** When stratified by M stage, M1 stage (n = 20) showed significantly lower expression of MT2A than M0 stage (n = 45). **E** When stratified by N stage, N1 stage (n = 22) showed significantly lower expression of MT2A than N0 stage (n = 28). **F** When stratified by TNM stage, stage III (n = 34) showed no significant expression of MT2A than stage I/II (n = 31), p = 0.1363. Scale bar = 100 μm. Can, cancer; Para, paracancerous; ns, no significance. *p < 0.05, **p < 0.01 and ***p < 0.001

To explore the clinicopathological characteristics of MT2A, we examined the expression of MT2A in our clinical samples. Sixty-five pairs of CRC samples involving cancer tissue and paracancerous tissue were collected and examined by IHC (Fig. 2A). Consistent with the aforementioned data, MT2A was significantly downregulated in cancer tissues compared to paracancerous tissues (Fig. 2B). We divided the patients into subtypes by clinicopathological characteristics. Our data showed that the expression of MT2A was not associated with T stage and TNM stage but was associated with M stage, and N stage in CRC patients (Fig. 2C–F). Thus, these results showed that MT2A is downregulated in CRC tissues and correlates with certain clinicopathological characteristics.

### MT2A inhibits cell proliferation and migration of CRC cells

**Fig. 3:**
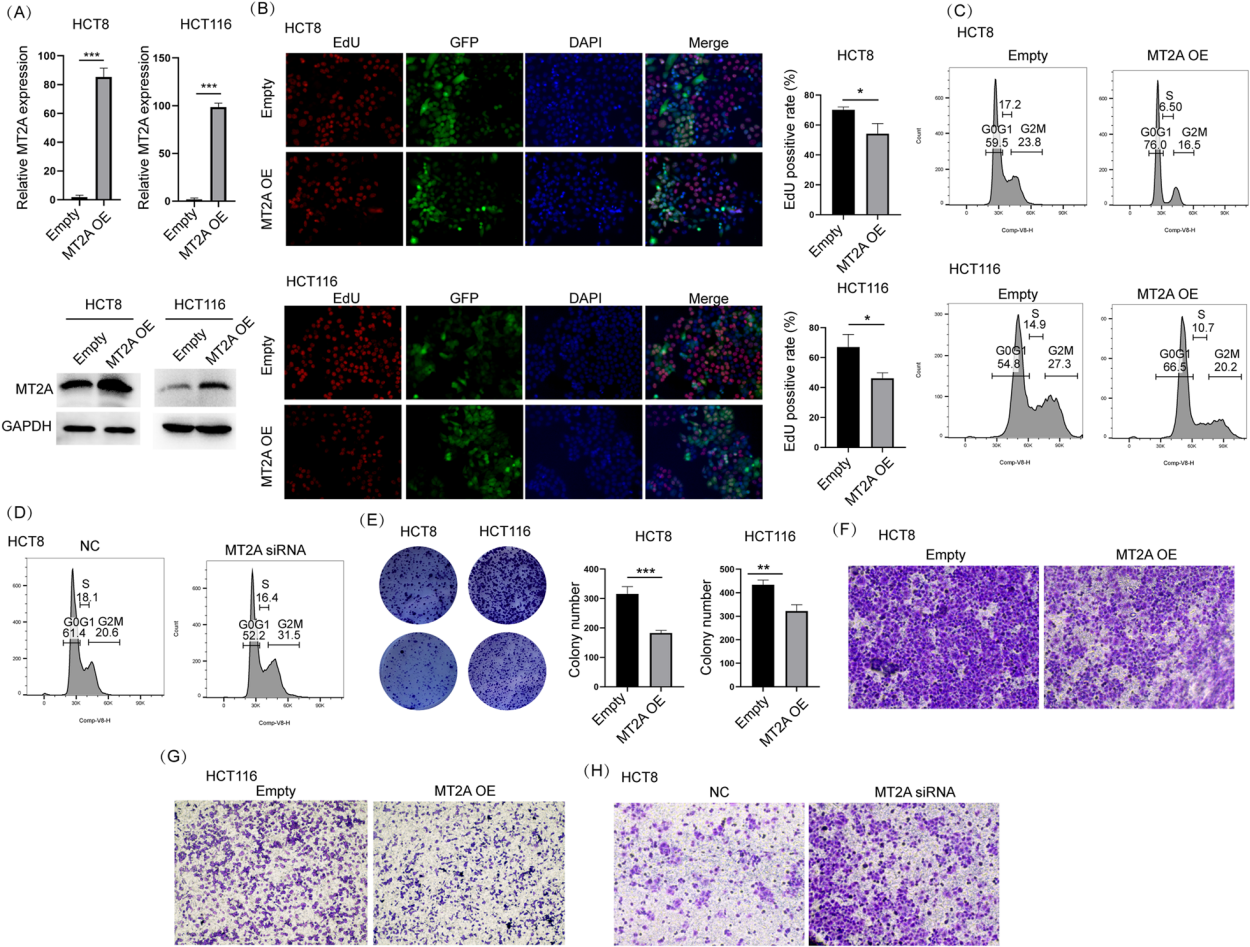
Overexpression of MT2A inhibits cell proliferation and migration of CRC cells. **A** A lentiviral vector was used to overexpress MT2A in HCT8 and HCT116 cells. The expression level of MT2A was examined by qRT–PCR and Western blot analyses, which confirmed that the MT2A lentiviral vector successfully overexpressed MT2A in HCT8 and HCT116 cells. **B** An EdU assay was used to evaluate tumor cell growth. MT2A overexpression significantly reduced the EdU-positive rate compared to control HCT8 and HCT116 cells. GFP expression indicated successful lentiviral transduction into both cell lines. **C** Flow cytometry was used to compare cell cycle distribution between empty and MT2A overexpression group in HCT8 and HCT116 cells. **D** Flow cytometry was used to analyze cell cycle distribution in HCT8 cells after knockdown of MT2A. **E** A colony formation assay was used to evaluate the effect of MT2A expression on colony formation ability, and the data showed that MT2A overexpression significantly inhibited colony formation in HCT8 and HCT116 cells. **F**, **G** Transwell assays showed that MT2A overexpression significantly inhibited cell migration compared to control HCT8 and HCT116 cells. 5 × 10^4^ cells were seeded in each well. **H** Transwell assays showed that knockdown of MT2A promoted migration of HCT8 cells. 1 × 10^4^ cells were seeded in each well. *OE* overexpression. *NC* negative control. *p < 0.05, **p < 0.01 and ***p < 0.001

To explore the function of MT2A in CRC cells, we used a lentiviral vector to overexpress MT2A in HCT8 and HCT116 cells. qRT–PCR and Western blot assays confirmed that MT2A was successfully overexpressed by lentivirus in both cell lines (Fig. 3A). As shown by the EdU assay, overexpression of MT2A significantly inhibited cell proliferation in both cell lines (Fig. 3B). Cell cycle distribution detected by flow cytometry showed that MT2A overexpression blocked cell cycle at G0G1 stage (Fig. 3C). Western blot showed that cyclin D1, a key cell cycle player regulating G1/S transition, was obviously inhibited by MT2A in both cells (Additional file 1: Figure S1A). To further demonstrate it, we used siRNA to knockdown MT2A in HCT8 which expressed high level of MT2A. Knockdown efficiency was verified by qRT-PCR (Additional file 1: Figure S1B). Cell cycle analysis showed that knockdown of MT2A improved cell cycle compared to negative control (Fig. 3D). Colony formation assay showed that overexpression of MT2A significantly inhibited the colony formation ability of HCT8 and HCT116 cells (Fig. 3E). Because the clinicopathological analysis indicated that higher expression of MT2A correlated with lower metastasis, we used a Transwell assay to examine whether MT2A influences the migration of CRC cells in vitro. The results showed that fewer cells migrated through the permeable membrane in the MT2A overexpression group than in the control group (Fig. 3F, G). Additionally, inhibition of MT2A improved migration of HCT8 cells (Fig. 3H). Thus, these data collectively demonstrated that MT2A inhibits the proliferation and migration of CRC cells in vitro.

### Overexpression of MT2A inhibits cell growth and liver metastasis of CRC cells in vivo

**Fig. 4:**
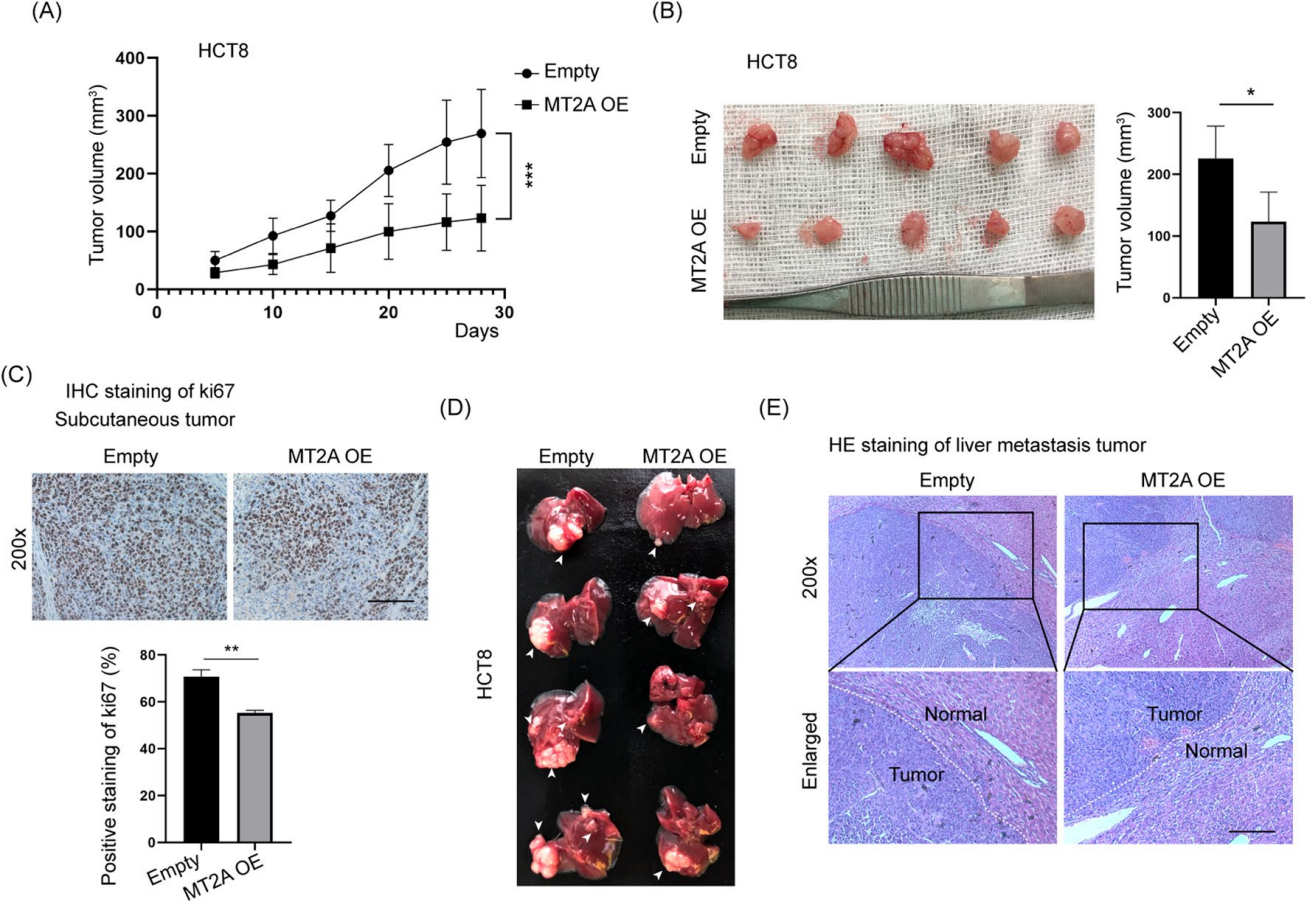
Overexpression of MT2A inhibits cell growth and liver metastasis of CRC cells in vivo. **A** HCT8 cells transfected with an empty vector or MT2A overexpression vector were subcutaneously injected into the right back of nude mice, which were examined for 4 weeks. The tumor volume was measured every 5 days, and the mice were sacrificed at Day 28 (n = 5). MT2A overexpression significantly decreased the tumor volume compared to empty vector control cells. **B** Tumors were collected, and tumor sizes were measured. The tumor size of MT2A-overexpressing cells was significantly smaller than that of control cells. **C** IHC staining of Ki67 was used to evaluate in vivo tumor growth, and MT2A overexpression significantly reduced Ki67 staining compared to the control. **D** A liver metastasis model was established by injection of HCT8 cells (2 × 10^6^ cells) under the spleen envelope. After 4 weeks, the mice were sacrificed, and the livers were dissected. The white arrow indicates the metastasis tumor. MT2A overexpression delayed liver metastasis of HCT8 cells. **E** The livers and metastatic tumors were embedded in paraffin, sectioned and analyzed. Compared to control cells, MT2A overexpression significantly reduced the compression of normal liver tissues. OE, overexpression. Scale, 100 μm

To verify the in vitro results, HCT8 cells were subcutaneously injected into nude mice, which were examined for 4 weeks. The tumor sizes were measured every 5 days, and the mice were sacrificed at Day 28 (Fig. 4A). The tumor size of MT2A-overexpressing cells was significantly smaller than that of control cells (Fig. 4B). Ki67 staining was used to evaluate cell growth in vivo, and the results showed that MT2A overexpression significantly inhibited Ki67 staining in subcutaneous tumors (Fig. 4C). These results demonstrated that overexpression of MT2A significantly delays tumor growth in vivo.

Because lower expression of MT2A correlated with M stage and N stage, we next examined whether MT2A influences liver metastasis of CRC cells. A liver metastasis model was established by injection of HCT8 cells (2 × 10^6^ cells) under the spleen envelope. After 4 weeks, the mice were sacrificed, and the livers were collected. Metastatic tumors were established in the liver, and a reduced tumor size was observed in cells transfected with MT2A lentivirus compared to control cells (Fig. 4D). H&E staining of the liver showed that more liver tissue was inundated with metastases from control cells compared to MT2A-overexpressing cells (Fig. 4E). These results demonstrated that overexpression of MT2A inhibits liver metastasis of CRC cells. Collectively, these data showed that overexpression of MT2A inhibits cell growth and liver metastasis of CRC cells in vivo.

### MT2A promotes Hippo signaling in CRC cells

**Fig. 5:**
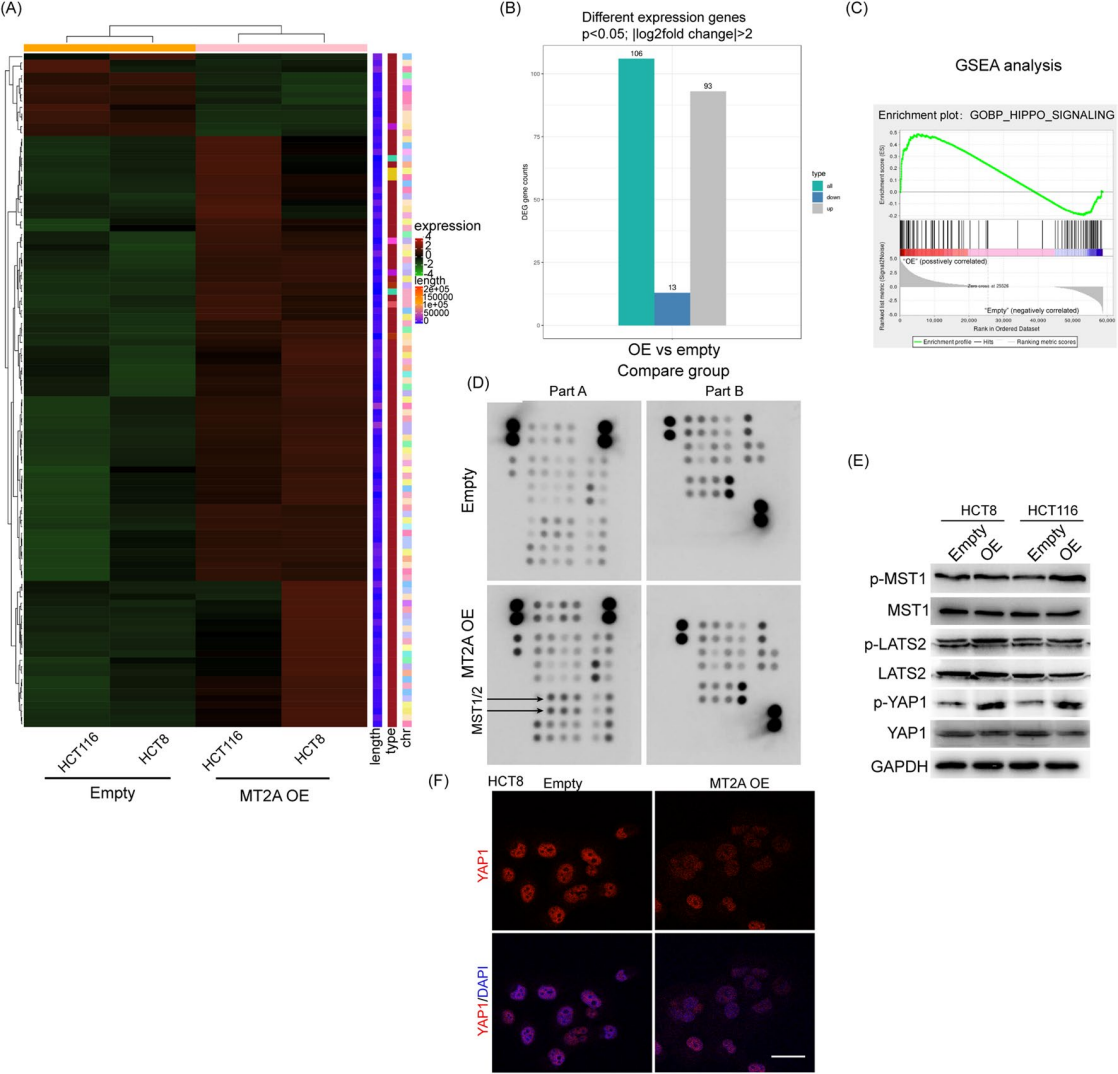
MT2A promotes Hippo signaling in CRC cells. **A** RNA sequencing was used to evaluate the gene expression level between MT2A-overexpressing and control HCT8 and HCT116 cells. **B** With a cutoff of p < 0.05 and |log_2_fold change|> 2, 106 genes were selected after MT2A overexpression in both cell lines, including 93 upregulated genes and 13 downregulated genes. **C** GSEA showed that MT2A overexpression significantly enriched Hippo signaling in HCT8 and HCT116 cells (normal p value < 0.05, NES > 1.0). **D** We used a Proteome Profiler Array Human Phospho-Kinase Array to identify the key variations in phosphokinases after transfection of MT2A. This array is a rapid and sensitive product used to detect relative levels of phosphorylation of 43 kinase phosphorylation sites and GAPDH protein. The A-B5 and B6 sites represent the expression levels of p-MST1 and p-MST2, respectively. MT2A overexpression promoted the phosphorylation of MST1 and MST2. **E** In HCT8 and HCT116 cells, the expression levels of p-MST1, MST1, p-LATS2, LATS2, p-YAP1, and YAP1 were determined by Western blot analysis. MT2A overexpression increased the phosphorylation of p-MST1, p-LATS2 and p-YAP1 in both cell lines. **F** Immunofluorescence staining of YAP1 showed that MT2A overexpression inhibited nucleus location of YAP1 in HCT8 cells. *OE* overexpression. Scale bar = 5 μm

To explore the underlying mechanism of MT2A in CRC cells, we used transcriptome sequencing to compare the overall expression differences between the MT2A overexpression and control groups in HCT8 and HCT116 cells. The heatmap showed that overexpression of MT2A led to many genes with dysregulated expression (Fig. 5A). Overexpression of MT2A in both cell lines resulted in 93 upregulated genes and 13 downregulated genes according to a cutoff of p < 0.05 and |log_2_fold change|> 2 (Fig. 5B). Gene set enrichment analysis (GSEA) analysis showed that overexpression of MT2A significantly correlated with Hippo signaling in CRC cells (Fig. 5C). The core components of Hippo signaling include Mst1/2 (Hippo in Drosophila), which phosphorylates LATS1/2 (Warts), leading to the phosphorylation of the YAP and TAZ transcription cofactors. To screen the core phosphokinases regulated by MT2A, we used a Proteome Profiler Array to identify the key variations in phosphokinases after transfection of MT2A. Interestingly, we found that overexpression of MT2A increased the expression of phospho-MST1/2 (Fig. 5D). As the functions of MST1 and MST2 or LATS1 and LATS2 were comparable in Hippo signaling, we only examined MST1 and LATS2 in the following study. Western blot assays confirmed that phospho-MST1 was activated by MT2A in HCT8 cells and HCT116 cells, and as expected, phospho-LATS2 was also increased by overexpression of MT2A, leading to activation of YAP1 (Fig. 5E). Normally, Hippo pathway is inactive, YAP is unphosphorylated and localized in the nucleus. To verify that whether MT2A inhibited the nucleus location of YAP, we used immunofluorescence staining of YAP in HCT8 cells. The result showed that MT2A overexpression could obviously inhibit nucleus location of YAP1 (Fig. 5F). In addition, the expression of TEA Domain transcription factor, such as TEAD1 or TEAD2, which are the key transcription factors of Hippo signaling, were not influenced by MT2A overexpression (Additional file 1: Figure S1C, D). Thus, our data demonstrated that overexpression of MT2A promotes phospho-MST1 and LATS2, resulting in inhibition of YAP1 in CRC cells.

### Inhibition of MST1/2 rescues the effect of MT2A on CRC cells

**Fig. 6:**
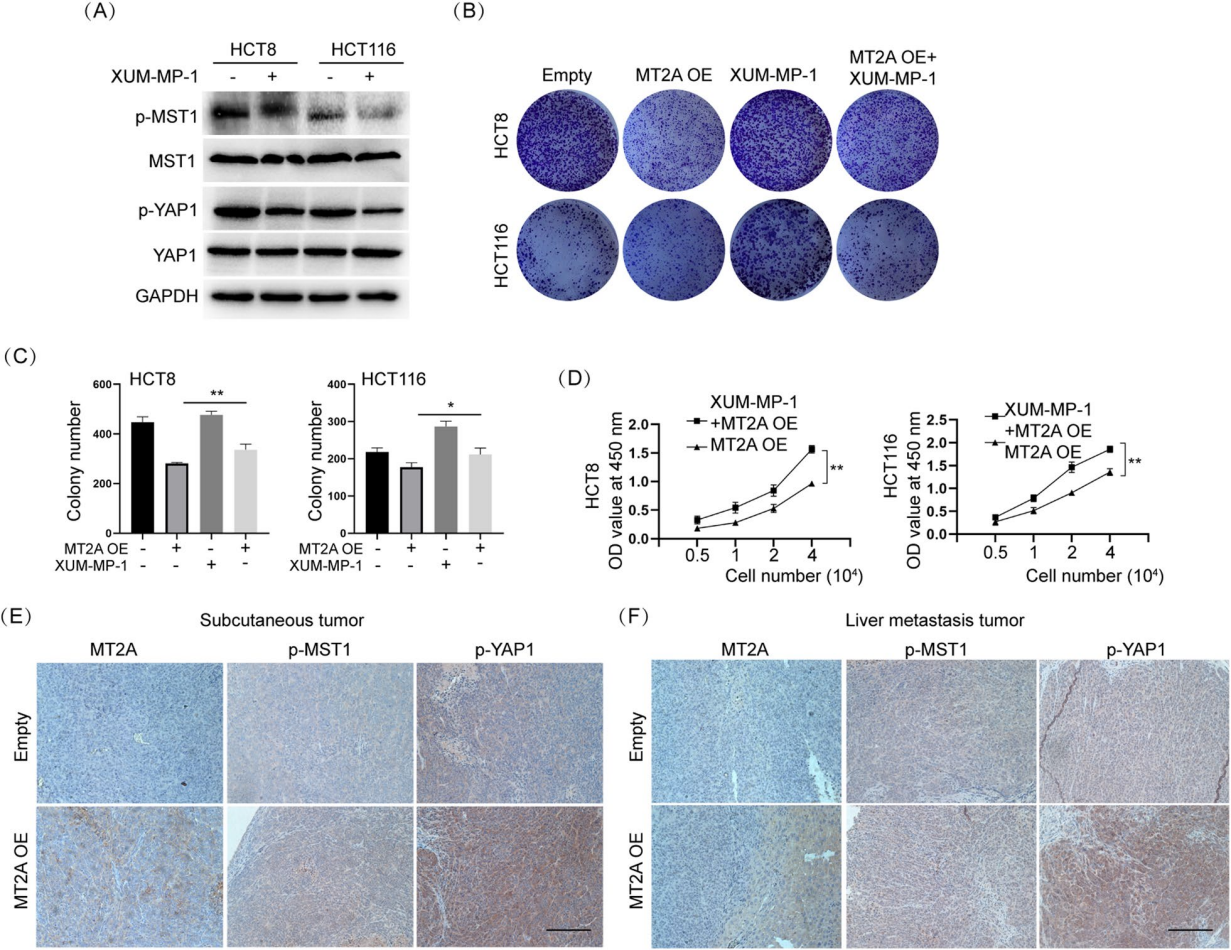
Inhibition of MST1/2 rescues the effect of MT2A on CRC cells. **A** XUM-MP-1 is a specific inhibitor of MST1/2. 2 μM XUM-MP-1 was added and cultured for 6 h. The results showed that both p-MST1 and p-YAP1 were significantly inhibited in HCT8 and HCT116 cells. **B**, **C** Colony formation assays showed that XUM-MP-1 promoted colony formation in HCT8 and HCT116 cells as well as rescued the inhibitory effect of MT2A overexpression. **D** The CCK-8 assay showed that XUM-MP-1 significantly increased the cell proliferation rate of MT2A-overexpressing cells. **E** IHC staining of MT2A showed that the MT2A overexpression group consistently overexpressed MT2A in subcutaneous tumors. P-MST1 and p-YAP1 were upregulated in the MT2A overexpression group compared to the control group. **F** Consistent with subcutaneous tumors, IHC staining of liver metastasis tumors showed that MT2A overexpression increased the expression of MT2A, p-MST1 and p-YAP1. Scale bar = 100 μm, OE, overexpression. *p < 0.05, **p < 0.01

To examine whether inhibition of Hippo signaling rescues the effect of MT2A on CRC cells, we used XMU-MP-1, a specific inhibitor of MST1/2, to inhibit the phosphorylation of MST1/2. p-MST1 was obviously inhibited by it in HCT8 and HCT116 cell in control group and MT2A overexpression group (Fig. 6A and Additional file 1: Figure S1E). Our results showed that treatment with XMU-MP-1 promoted colony formation in HCT8 and HCT116 cells, which was consistent with previous reports (Fig. 6C, D). Importantly, XMU-MP-1 largely attenuated the effect of MT2A on colony formation and cell proliferation in HCT8 and HCT116 cells (Fig. 6D, E). Thus, these data demonstrated that inhibition of MST1/2 rescues the effect of MT2A on CRC cells.

To verify that the Hippo/YAP1 axis was also regulated by MT2A in vivo, we examined the expression of MST1, YAP1 and MT2A in animal samples. In the subcutaneous tumor samples, IHC staining showed that overexpression of MT2A promoted the expression of phosphorylated MST1 and YAP1 compared to the control (Fig. 6F). A similar result was found in liver metastasis samples, in which MT2A overexpression increased the phosphorylation levels of MST1 and YAP1 compared to the control (Fig. 6G). Thus, these results demonstrated that phosphor-MST1/YAP1 is positively associated with MT2A and that inhibition of phosphor-MST1 by pharmacological methods largely rescues the effect of MT2A in CRC cells.

### Discussion

Colorectal cancer, as one of the deadliest cancers, is the most common malignant tumor with liver metastasis and a poor prognosis. Some CRC patients have liver metastasis at the time of diagnosis, preventing the ability to treat CRC with surgery. Therefore, it is of great significance to explore the mechanism of liver metastasis of CRC. Increasing evidence indicates that metallothioneins play an important role in tumor growth, differentiation, angiogenesis, metastasis, microenvironmental remodeling, immune escape and drug resistance, and they have potential as biomarkers for tumor diagnosis and prognosis. There are four main isomers of MTs, including MT1, MT2, MT3 and MT4. Among them, the role of MT2 in colorectal cancer has not been widely studied. First, we found that MT2A was downregulated in colorectal cancer using TCGA, GSE and Oncomine databases. These findings were verified in 65 pairs of cancer and adjacent tissues in patients with CRC. These results were consistent with those reported by Meng et al. [16] and Arriaga et al. [13]. However, other studies have shown that MT2A is upregulated in human colorectal adenocarcinoma HT29 cells [12], and the interaction between pFADD and MT2A inhibits the apoptosis of Colo 205 cells and induces cell proliferation [14]. There is a complex relationship between MTs and cancers. For example, MT2A is upregulated in osteosarcoma, breast cancer and prostate cancer [17–19], with carcinogenic roles, while it is downregulated in gastric carcinoma, hepatoma and lung cancer [20–22], with cancer suppression roles. Moreover, MT2A expression is controversial in lung cancer. A recent study has found that high expression of MT2A is associated with poor prognosis of lung adenocarcinoma [23]. However, a previous study has found that MT2A expression levels in lung cancer tissues are significantly downregulated compared to paracancerous tissues [21]. In addition, another study has demonstrated that the upregulation of MT2A is associated with poor survival in patients with non-small-cell lung cancer [24]. These results suggest that the expression of MTs is related to multiple factors, such as different tumor types, tumor differentiation status, environmental stimulation and gene mutation.

In patients with CRLM, the content of MTs in liver metastases is significantly lower than that in surrounding normal liver tissues [25]. Immunohistochemistry has been used to detect the expression of MT in 117 cases with CRLM; and the results showed that there were 103 negative cases and 14 low or moderate cases, while peripheral hepatocytes and stromal cells were positive [26]. The present study showed that the MT2A expression level in colorectal cancer was correlated with tumor M stage. Subsequently, MT2A overexpression inhibited the proliferation and metastasis of human colorectal adenocarcinoma HCT8 and HCT116 cells in vitro. Animal models with liver CRLM also confirmed that overexpression of MT2A inhibited liver metastasis. At present, the specific mechanism by which MT2A affects liver metastasis of CRC is not clear. Transcriptome sequencing was used to detect differentially expressed genes in MT2A-overexpressing cells and control cells, and the results showed that MT2A overexpression was significantly correlated with the Hippo signaling pathway.

**Fig. 7:**
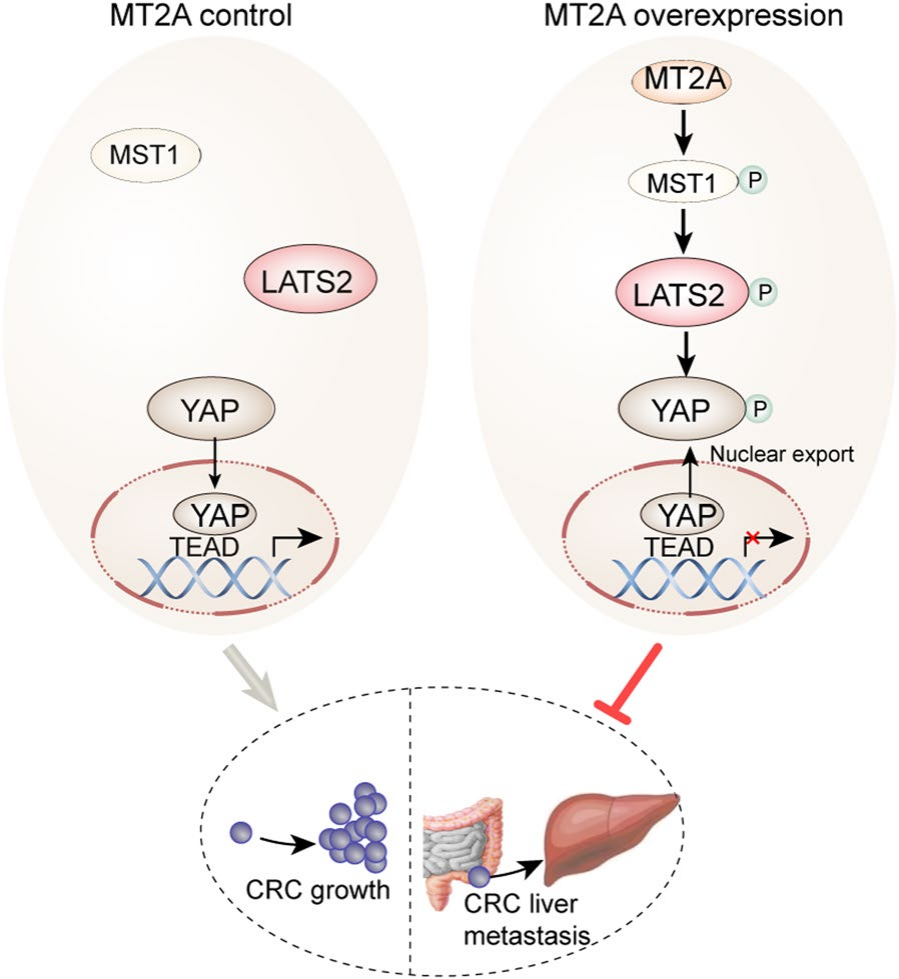
Schematic of MT2A overexpression mediated inhibition of cell growth and liver metastasis in CRC cells. Normally, Hippo pathway is inactive, YAP is unphosphorylated and localized in the nucleus to bind with TEAD and activation of gene transcription, but when MT2A overexpressed, MST1 phosphorylates LATS2, and then phosphorylates YAP1. Phosphorylated YAP1 is exported from nucleus and inhibits gene transcription

The Hippo pathway is dysregulated in a variety of tumors, including CRC, and it affects a variety of phenotypes through direct and indirect effects, such as cell proliferation, metabolic reprogramming, angiogenesis, invasion, metastasis, cancer stem cells, inflammation and immunosuppression [27]. Many studies have confirmed the role of the Hippo signaling pathway in colorectal cancer metastasis, and the specific mechanism, for instance, may be that upregulated SCRIB expression promotes YAP phosphorylation, thereby inhibiting the Hippo pathway [28]. USP2-AS1 decreases phosphorylated YAP and increases the total level of YAP1, promoting the proliferation and metastasis of CRC cells [29]. Moreover, downregulation of RASAL2 promotes YAP1 phosphorylation, cytoplasmic retention and ubiquitination, thereby activating the Hippo pathway through the LATS2/YAP1 axis and playing a role in the development and metastasis of CRC [30]. In the present study, increased expression of phosphorylated Mst1, LATS2 and YAP1 was observed in CRC cells overexpressing MT2A, and similar results were also observed in subcutaneous CRC tumor and liver metastasis tissues overexpressing MT2A according to immunohistochemical staining. The specific inhibitor of MST1/2 (XMU-MP-1) inhibited the MT2A overexpression-induced phosphorylation of MST1, LATS2 and YAP1, thus removing the inhibition of the Hippo pathway. In summary, our study found that MT2A is expressed at low levels in CRC and correlates with M stage, and we demonstrated that MT2A mediates CRC cell proliferation and liver metastasis through the MST1/LATS2/YAP1 signaling pathway (Fig. 7). Although the MT2A expression level in CRC may be related to tumor heterogeneity and further studies are needed to explore the reasons for this phenomenon, our results suggested that MT2A is a promising therapeutic target for colorectal cancer.

## Supplementary Information

**Additional file 1: Figure S1.** **A** Western blot assay showed that overexpression of MT2A decreased the expression of cyclin D1 in HCT116 and HCT8 cells. **B** siRNA successfully knockdown MT2A in HCT8 cells. **C** Using data from RNA-seq TEADs RNA was not influenced by MT2A overexpression. **D** TEAD1 and TEAD2 protein was not influenced by MT2A in HCT8 and HCT116 cells. **E** XMU-MP-1 inhibited p-MST1 in HCT8 and HCT116 cells with overexpression of MT2A. *OE* overexpression. ***p < 0.001.

## Data Availability

All datasets presented in this study are included in the article/additional files.
