# Supplementary figures and images for: Metallothionein 2A (MT2A) controls cell proliferation and liver metastasis by controlling the MST1/LATS2/YAP1 signaling pathway in colorectal cancer
Source: Cancer Cell Int. 2022 May 31;22:205. doi: 10.1186/s12935-022-02623-w (PMC9158144; doi:10.1186/s12935-022-02623-w)

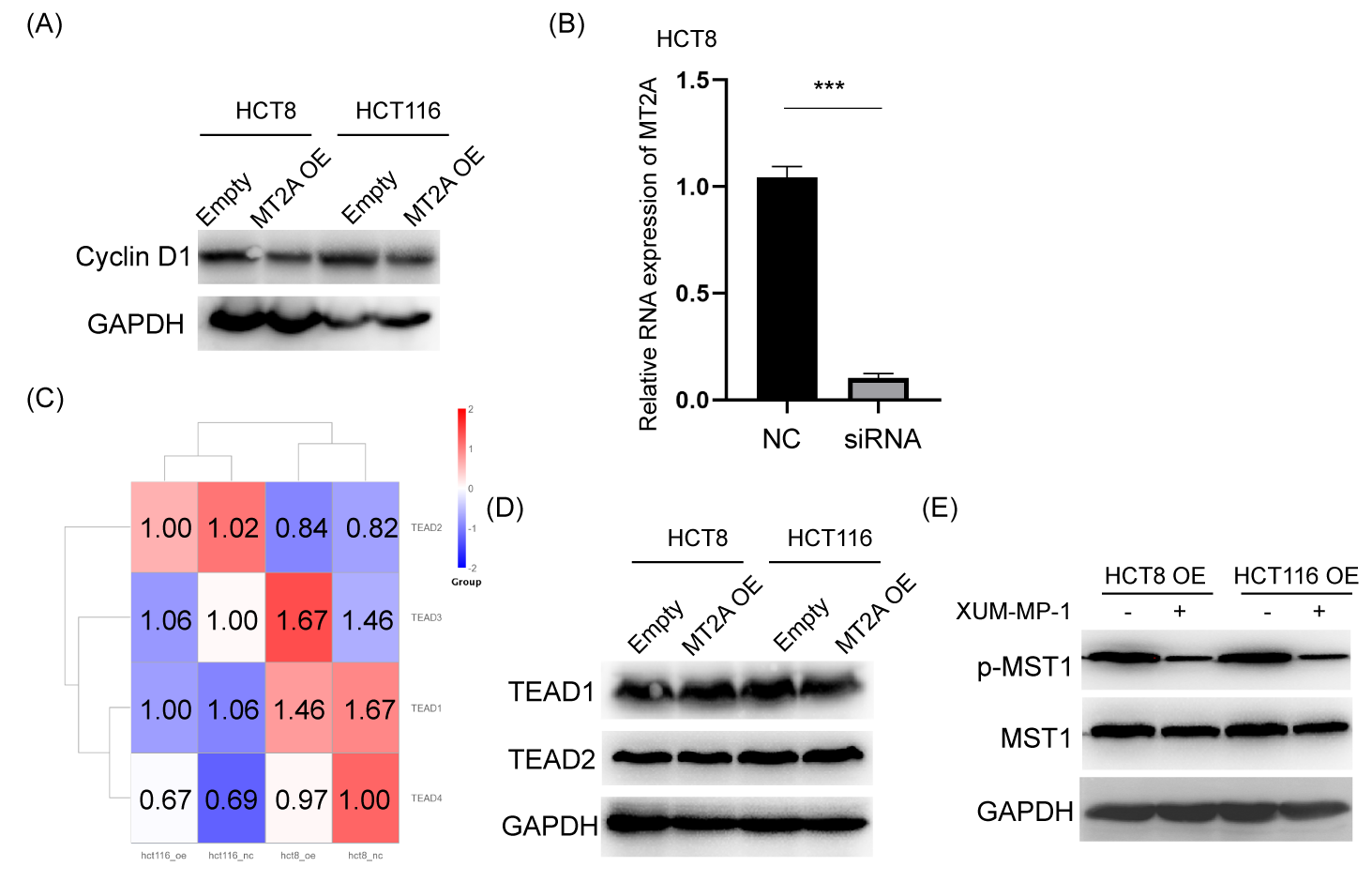

Supplement: Supplementary file 1 — Additional file 1: Figure S1. A Western blot assay showed that overexpression of MT2A decreased the expression of cyclin D1 in HCT116 and HCT8 cells. B siRNA successfully knockdown MT2A in HCT8 cells. C Using data from RNA-seq TEADs RNA was not influenced by MT2A overexpression. D TEAD1 and TEAD2 protein was not influenced by MT2A in HCT8 and HCT116 cells. E XMU-MP-1 inhibited p-MST1 in HCT8 and HCT116 cells with overexpression of MT2A. OE overexpression. ***p < 0.001. [file 12935_2022_2623_MOESM1_ESM.tif]
